# Case Report: Ureteral double-J stent placement: a rare case of ureteral perforation complicated by periureteral abscess

**DOI:** 10.3389/fsurg.2026.1890796

**Published:** 2026-07-20

**Authors:** Tao Zhou, Lei Yao

**Affiliations:** 1 Department of Urology, The Second Affiliated Hospital of Zhejiang Chinese Medical University, Hangzhou, Zhejiang, China; 2 Zhejiang Provincial Key Laboratory of Sexual Function of Integrated Traditional Chinese and Western Medicine, Hangzhou, Zhejiang, China; 3 Zhejiang Provincial Key Laboratory of Traditional Chinese Medicine, Hangzhou, Zhejiang, China

**Keywords:** case report, double-J stent insertion, periureteral abscess, ureteral calculi, ureteral perforation

## Abstract

**Background:**

This case report presents the first documented instance of ureteral perforation complicated by a periureteral abscess following double-J stent placement. The diagnostic and therapeutic experience is summarized herein.

**Case presentation:**

An 83-year-old female with type 2 diabetes mellitus and ureteral calculi underwent ureteral stent insertion for stone-induced obstruction. Subsequently, she developed ureteral perforation accompanied by a periureteral abscess. Management involved stent removal, left percutaneous nephrostomy, ultrasound-guided abscess drainage, and targeted antibiotic therapy. Eventually, the patient's symptoms resolved, the abscess was completely absorbed, and no recurrence was observed during follow-up.

**Discussion:**

Double-J ureteral stent insertion can lead to numerous life-threatening complications. Improper stent placement may result in ureteral perforation and subsequent periureteral abscess formation. This risk is particularly elevated in elderly diabetic patients with chronic infection due to ureteral calculi, as the ureteral wall is fragile and prone to perforation during retrograde catheterization. Percutaneous nephrostomy may represent a more appropriate strategy for relieving obstruction in such cases. If stent insertion is performed, forceful maneuvers must be avoided during the procedure, the procedure should be performed under fluoroscopic guidance and early postoperative CT imaging is recommended to confirm proper stent positioning. Ureteral perforation typically heals spontaneously without surgical repair. Once a periureteral abscess develops, prompt percutaneous drainage is essential, as antibiotic therapy alone has limited efficacy.

## 1 Introduction

Ureteral double-J stent placement has become a common procedure for the management of various urological conditions. However, with the increasing use of ureteral stents, the incidence of complications associated with their placement has also risen (1). Early complications of double-J ureteral stent placement include stent-related discomfort, vesicoureteral reflux, and ureteral smooth muscle spasm (2). In contrast, the most common late complications are stent migration, encrustation, and fragmentation (3, 4). This report documents a case of ureteral perforation complicated by periureteral abscess formation following ureteral stent placement. To the best of our knowledge, this is the first reported case of ureteral perforation with periureteral abscess induced by double-J stent placement. This case report aims to share our experience in managing this rare complication of ureteral perforation accompanied by periureteral abscess formation.

## 2 Case presentation

A case of an 83-year-old female patient is reported here. She was admitted due to “low-grade fever for 6 days.” The patient had a history of type 2 diabetes mellitus for over 30 years, complicated by diabetic foot. Prior to admission, she had received continuous anti-infective therapy with meropenem at an external hospital for 6 days without significant improvement. Upon admission, computed tomography (CT) revealed “a left upper ureteral calculus with mild hydronephrosis.” Laboratory findings showed a urine leukocyte count of 3,395 cells/*μ*L, a blood leukocyte count of 7.4 × 10⁹/L, a neutrophil percentage of 85.3%, a platelet count of 67 × 10⁹/L, a high-sensitivity C-reactive protein (hs-CRP) level of 20.6 mg/L, and a procalcitonin level of 1.71 ng/mL (Supplement Table 1). Serum creatinine was within normal limits. Considering the patient's advanced age and the definitive diagnosis of ureteral calculus with hydronephrosis and infection, a “transurethral cystoscopic ureteral stent placement” was performed retrogradely without fluoroscopic guidance, using a 0.028-inch hydrophilic guidewire and one F5 double-J (D-J) stent was inserted.

**Figure 1:**
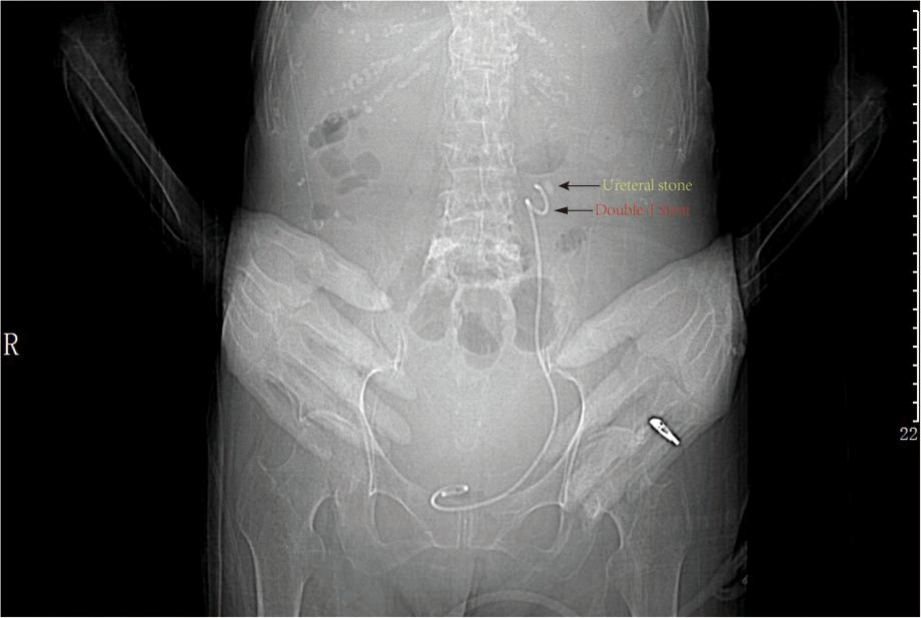
KUB radiography taken on the 1st postoperative day.

**Figure 2:**
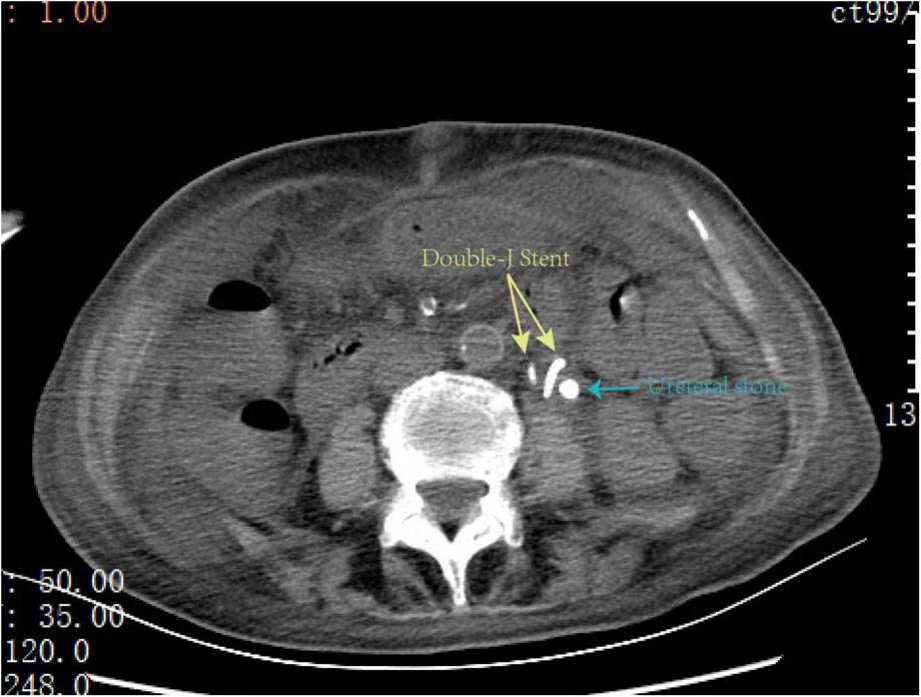
Abdominal CT image on the 1st postoperative day.

On the first postoperative day, the patient developed persistent left lower back pain, low-grade fever (38.0 °C), a urine leukocyte count of 548 cells/μL, a blood leukocyte count of 6.6 × 10⁹/L, a neutrophil percentage of 83.4%, a platelet count of 79 × 10⁹/L, and an hs-CRP level of 44.2 mg/L (Supplement Table 1). CT scan demonstrated a round, high-density shadow in the left upper ureter, approximately 6 mm in diameter, with dilation of the renal pelvis and proximal ureter. The D-J stent was not visualized within the ureter; its proximal end was located medial to the ureter, and there was no diffuse urinary extravasation, indicating that the ureteral stent had not bypassed the obstructed segment (Figures 1, 2). Anti-infective therapy with meropenem injection was continued, due to persistent pyrexia and elevated hs-CRP. On postoperative day 4, the patient experienced recurrent low-grade fever. Blood culture results indicated “Candida infection.” Urine leukocyte count was 1,095 cells/μL, blood leukocyte count was 6.3 × 10⁹/L, neutrophil percentage was 88.3%, platelet count was 63 × 10⁹/L, and hs-CRP level was 100.38 mg/L (Supplement Table 1). Consequently, therapy was switched to micafungin sodium powder for injection combined with cefoperazone sodium and sulbactam sodium for anti-infection.

**Figure 3:**
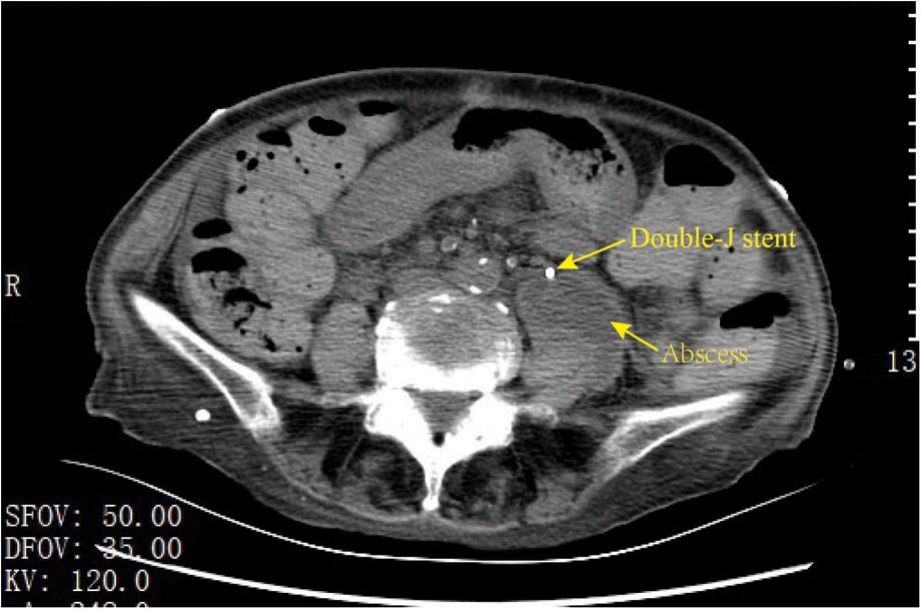
Abdominal CT image on the 12th postoperative day.

**Figure 4:**
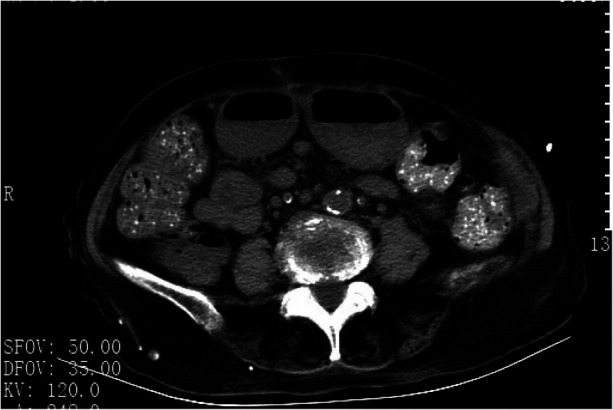
Abdominal CT image obtained two months after percutaneous abscess drainage.

CT re-examination on postoperative day 12 revealed an abscess formation adjacent to the left psoas major muscle surrounding the ureter (Figure 3). Laboratory results showed a urine leukocyte count of 656 cells/μL, a blood leukocyte count of 6.7 × 10⁹/L, a neutrophil percentage of 85.8%, a platelet count of 107 × 10⁹/L, and an hs-CRP level of 61.1 mg/L (Supplement Table 1). The following day, ureteral stent removal was performed; however, a left percutaneous nephrostomy was not performed until one week later. Post-nephrostomy, the patient's lower back pain improved, but intermittent low-grade fever persisted. Laboratory results showed a urine leukocyte count of 212 cells/μL, a blood leukocyte count of 6.0 × 10⁹/L, a neutrophil percentage of 70.5%, a platelet count of 104 × 10⁹/L, and an hs-CRP level of 59.5 mg/L (Supplement Table 1). After three more weeks of continued anti-infective therapy, the abscess showed no significant reduction. Therefore, ultrasound-guided abscess puncture and drainage was performed, followed by anti-infective therapy with ceftazidime. The patient became afebrile immediately after the procedure. The aspirated pus culture was negative for both bacterial and fungal growth. Post-drainage laboratory results showed a urine leukocyte count of 1,033 cells/μL, a blood leukocyte count of 3.3 × 10⁹/L, a neutrophil percentage of 64.1%, a platelet count of 79 × 10⁹/L, and an hs-CRP level of 21.9 mg/L (Supplement Table 1). After one additional week of treatment, the patient's symptoms improved, and she was discharged. CT re-examination performed two months after the abscess puncture and drainage showed that the abscess had resolved (Figure 4). During a six-month follow-up period, the patient remained afebrile and reported no recurrence of flank pain or lower urinary tract symptoms. A repeat CT scan at the final follow-up confirmed the complete resolution of the abscess and stable decompression of the collecting system.

## 3 Discussion

Currently in clinical practice, D-J stent placement is the standard operational procedure following endourological interventions (5). Additionally, D-J stent placement is commonly utilized in the management of gynecologic tumors, trauma, retroperitoneal fibrosis, renal transplantation, hydronephrosis during pregnancy, and extracorporeal shock wave lithotripsy (6). However, it may lead to severe complications, including malposition, migration, encrustation, calcification, subcapsular hematoma, and infection (1). Geavlete et al, in a comprehensive study, reported that among the common complications of D-J stent placement, 0.3% were attributed to stent malposition. They further noted that proximal stent migration occurred in 0.9% of cases, while distal migration occurred in 0.7% of all cases. Ureteral stent obstruction with poor drainage was observed in 1.85% of patients, and bladder irritative symptoms were present in 32.7% of patients. The incidence of hematuria was 10.38%, and the diagnosis rate of urinary tract infection was 14.8%. The rate of stent encrustation and calcification was 1.66% (7). M. Resorlu reported a case of ureteral perforation without concomitant periureteral abscess, which was successfully managed by re-inserting a ureteral stent (8).A case of large subcapsular hematoma of the liver following ureteral stent placement was reported by Blas (9).

In this report, we present a case of ureteral perforation complicated by periureteral abscess formation resulting from improper ureteral stent placement. The patient was an elderly female with diabetes mellitus who initially presented with hydronephrosis and infection caused by a ureteral calculus. During the treatment course, complete clinical cure was achieved following percutaneous nephrostomy, stent removal, and abscess puncture drainage, with subsequent spontaneous healing of the ureteral wall. The causes of perforation were considered as follows: firstly, fragility of the ureteral wall, potentially attributable to (1) a two-week history of calculus impaction leading to ureteral mucosal inflammation, and (2) advanced age and concomitant diabetes mellitus contributing to increased ureteral wall vulnerability; secondly, increased resistance during retrograde catheterization due to calculus obstruction, resulting in a tendency for the guidewire to buckle below the calculus; thirdly, forceful manipulation during stent placement; fourthly, the absence of fluoroscopic guidance may have been a critical contributing factor. During retrograde insertion in the presence of an impacted calculus, the guidewire tends to buckle or deviate below the stone, especially without real-time imaging to confirm its intraluminal position. Following ureteral perforation, urine extravasation along the ureteral stent, combined with the patient's weakened immune status and a duration of antimicrobial therapy exceeding two weeks, predisposed the patient to fungal infection, thereby creating favorable conditions for abscess formation. Despite relief of urinary tract obstruction, given the patient's advanced age, compromised immunity, and concurrent fungal infection, treatment with systemic antimicrobial therapy alone was insufficient to achieve clinical cure; abscess puncture drainage was also required. Therefore, timely percutaneous drainage of purulent fluid is essential when ureteral perforation caused by stent placement is complicated by periureteral abscess formation. Ureteral perforation resulting from improper stent placement may heal spontaneously without the need for repeat stent insertion.

Besides critical analysis of our case reveals a significant management delay. Despite CT confirmation of stent malposition on postoperative day 1, the stent was not removed until day 13. This 13-day period of leaving a perforated, misplaced foreign body in a patient with an active urinary tract infection and diabetes mellitus directly contributed to the progression of local inflammation into a periureteral abscess and fungemia. This oversight constitutes a major clinical error and highlights the absolute necessity of prompt intervention.

As the population ages and healthcare resources continue to expand, the incidence of such cases may gradually increase. This report aims to provide clinical experience for future reference. As a single case report, this study is inherently limited by its nature, including the inability to establish causal relationships, the potential for overinterpretation, and publication bias. Consequently, the methods described herein require further validation in future clinical practice.

## 4 Reflections on this case

Selection of the method for relieving obstruction: In elderly patients with ureteral calculi and prolonged infection, particularly those with compromised ureteral wall integrity due to chronic inflammation and diabetes, PCN may serves as a better choice for urinary diversion than ureteroscopic stent placement for relieving obstruction. Chronic inflammatory stimulation and age-related tissue degeneration may render the ureteral wall more fragile, thereby increasing the risk of perforation during stent insertion. Early localization following stent placement: If ureteral stent placement is selected, intraoperative localization of the stent and early postoperative imaging assessment are critical to prevent disease progression. Management following failed stent placement: If ureteral stent placement is unsuccessful and results in ureteral perforation, conversion to percutaneous nephrostomy should be performed promptly. Simultaneously, the ureteral stent should be removed immediately and without delay to prevent it from serving as a nidus of infection. Our case tragically exemplifies the consequences of failing to adhere to this principle. Spontaneous healing of ureteral perforation: Ureteral perforation caused by stent placement does not require surgical repair and may heal spontaneously following urinary decompression. Management of periureteral abscess: Abscess formation surrounding a ureteral perforation should be managed with early puncture drainage, as conservative treatment alone is often insufficient. Avoidance of forceful manipulation during stent placement: If significant resistance is encountered during stent insertion, forceful manipulation should be avoided. The stent should be withdrawn, and the guidewire position should be readjusted until the stent can be advanced without notable resistance.

## Data Availability

The original contributions presented in the study are included in the article/Supplementary Material, further inquiries can be directed to the corresponding author.
